# Structure, function and cell dynamics during chaetogenesis of abdominal uncini in *Sabellaria alveolata* (Sabellariidae, Annelida)

**DOI:** 10.1186/s40851-016-0037-4

**Published:** 2016-01-08

**Authors:** Ekin Tilic, Thomas Bartolomaeus

**Affiliations:** Institute of Evolutionary Biology and Ecology, Rheinische Friedrich Wilhelms Universität Bonn, An der Immenburg 1, 53121 Bonn, Germany

**Keywords:** TEM, cLSM, Chaetogenesis, Hooked chaetae, Polychaetes, Sedentaria, Functional morphology

## Abstract

**Background:**

Dynamic apical microvilli of a single cell, called the chaetoblast, inside an ectodermal invagination form the template of annelid chaetae. Changes in the pattern of microvilli are frozen in time by release of chitin, such that the structure of the definitive chaeta reflects its formation. Cellular interactions during chaetogenesis also influence the structure of the chaeta. Analysing chaetogenesis allows for testing hypotheses on the homology of certain chaetal types. We used this approach to test whether the unusual uncini in *Sabellaria alveolata* are homologous to apparently similar uncini in other annelid taxa.

**Results:**

Our study reveals unexpected details of sabellariid uncini, which mechanically reinforce the neuropodia enabling their use as paddles. The final structure of the chaeta is caused by pulses of microvilli formation and dynamic interaction between the chaetoblast and adjoining follicle cells. Cell dynamics during chaetogenesis of the uncini in *Sabellaria alveolata* exceeds by far that reported in previous studies on the formation of this type of chaetae.

**Conclusion:**

Despite the superficial similarity of uncini in sabellariids and other annelids, differences in structure and details of formation do not support the homology of this type of chaetae. Chaetogenesis of sabellariid uncini involves unexpected microvilli and cell dynamics, and provides evidence that interactions between cells play a larger role in chaetogenesis than previously expected. In addition to their function as anchors, uncini in Sabellaridae stabilize the paddle-shaped notopodia, as each uncinus possesses a long, thin rod that extends deeply into the notopodium. The rods of all uncini in a single row form a bundle inside the notopodium that additionally serves as a muscle attachment site and thus have a similar function to the inner chaeta (acicula) of errant polychaetes (Aciculata).

## Background

Chaetae are chitinous extracellular structures that are important diagnostic characters in Annelida [1, 2]. Chaetae are formed within an ectodermal invagination, the chaetal follicle, which consists of a terminal chaetoblast and a few follicle cells [3–5]. Each chaetoblast has an array of apical microvilli that are modified in time and space while chitin polymerizes alongside the microvilli. Controlled modification of the microvilli pattern, thus, gives the chaeta its final shape [4, 6–8]. Alterations in spatiotemporal patterns allow the formation of a plethora of different chaetal types that can range from highly complex compound hooked chaetae to simple capillaries. Chaetogenesis involves such an elaborate interplay of cellular instruments that genetic programming and regulation is necessary to establish consistent chaetal arrangement and structure within annelid species and supraspecific taxa [9, 10]. Given that genetic information underlies chaetogenesis, we assume that any hypothesis on the homology of chaetae can be tested. Identical formation processes are expected for homologous and structurally similar chaetae.

Hooked chaetae and uncini possess several small apical teeth giving the chaetae a saw- or rasp-shaped appearance when viewed from above. These teeth may or may not surmount a single large tooth. Small apical teeth and the main tooth, if present, are curved relative to the shaft which represents the main axis of the chaeta. Uncini and hooked chaetae are discriminated by the length of the shaft, although its length is an imprecise character that varies intra- and supraspecifically [11]. Studies into the chaetogenesis of the hooked chaetae and uncini in certain sedentary polychaetes has revealed that the structure of these chaetae actually results from a uniform formation process (Sabellidae and Serpulidae [11–13]; Arenicolidae [14, 15]; Maldanidae [10]; Psammodrilida [16], Terebellida [12, 17]; Oweniidae [18]; Siboglinidae [19]). One of the major conclusions of these studies is that substructures and course of formation support the homology hypothesis for hooked chaetae and uncini, at least for the taxa studied [20]. Sabellariidae, which possess uncini that are aligned in a transverse row at the outer rim of the abdominal notopodia, however, have not been included in such comparative studies to date. At least on the level of light microscopy, sabellariid uncini do not seem to differ from uncini of the other taxa studied so far.

In the present study, we have investigated the ultrastructure and chaetogenesis of abdominal uncini in *Sabellaria alveolata* (Sabellariidae). Assuming all uncini are homologous, one would expect significant similarities in chaetal ultastructure and formation. Although for epistemological reasons it is impossible to prove non-homology, recognizable differences in mode of chaetogenesis would not support the homology of sabellaridan uncini to those of other hemisessile and sessile annelids with hooked chaetae, but would rather allow alternative hypotheses for the position of Sabellariidae.

## Material and methods

### Animals

**Fig. 1:**
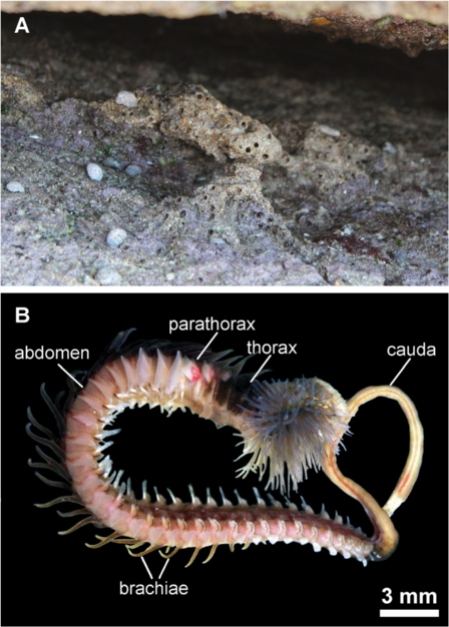
**a** Collection site of *Sabellaria alveolata*, showing the distinctive tubes under a rock crevice. **b** Habitus of *Sabellaria alveolata,* showing the different body regions

*Sabellaria alveolata* (Linnaeus, 1767) (Fig. 1) was collected in March 2013 in the rocky intertidal of Concarneau (Brittany, France). Here, *S. alveolata* occurs in dense colonies in sheltered rock crevices, building distinctive hard tubes from the sediment (Fig. 1a). The tubes were removed from the rocks with the help of a spatula and the animals were fixed in the field immediately after being removed from their tube.

### Light microcopy (LM), histology and 3D reconstruction

The specimens of *Sabellaria alveolata* used for the serial semi-thin sections and the 3D reconstruction was fixed in 1.25 % glutaraldehyde buffered in 0.05 M phosphate buffer with 0.3 M NaCl for 1.5–2 hours. The fixed animals were stored in the same buffer until they were postfixed in 1 % OsO_4_ for 45 min. The specimens were dehydrated in an acetone series right after the postfixation, transferred in propylene oxide and embedded in araldite. If necessary, specimens were sectioned into smaller pieces within the resin. Polymerization was started with BDMA (Benzyldimethylamine). A series of one micrometer sections were cut with a diamond knife (Diatome Histo Jumbo) on a Leica Ultracut S ultramicrotome, following the method described by Blumer et al. [21]. The sections were stained with toluidine blue (1 % toluidine, 1 % sodium-tetraborate and 20 % saccharose) and covered with a cover slip mounted with araldite. The semi-thin sections were analyzed with an Olympus microscope (BX-51) and photographed with an Olympus camera (Olympus cc12), equipped with the dot slide system (2.2 Olympus, Hamburg). Images were aligned using IMOD (Boulder Laboratories, [22]) and IMOD-align (http://www.evolution.uni-bonn.de/mitarbeiter/bquast/software).

3D modelling of the chaetae was performed using the software 3ds max 13. Histological images were imported as surface materials (discreet) and the chaetae were modeled using standard cylindrical objects. When necessary, these were modified as NURBS (Nonuniform rational B-Splines)-surfaces. The outline of the neuropodial torus was created using another NURBS surface.

Using the same method a second 3D model was constructed with the aligned TEM-images of the formative site. Here all of the studied developmental stages were modeled in order to visualize their topological position within the formative site.

Single chaetae analyzed using a confocal laser scanning microscope and Nomarsky differential interference contrast under an Olympus BX-51 microscope were isolated from pieces of PFA (1 h in 4 % paraformaldehyde) fixed specimens of *Sabellaria alveolata* by incubation in 5 % NaOH for 4–5 h. The chaetae were rinsed in distilled water, mounted on microscopical slides and examined.

### Confocal laser scanning microscopy (CLSM)

The specimens used for confocal laser scanning microscopy were fixed in 4 % paraformaldehyde for 1 h and later stored in 0.1 M PBS (phosphate buffered with saline) containing 0.01 % NaN_3_. The chaetigers were dissected to separate single parapodia. Isolated parapodia and segments were permeabilized in four 5-min changes of PBS with 0.1 % Triton X-100 (Fisher Scientific). The samples were then stained overnight in 4 °C with TRITC phalloidin at a dilution of 1:100. After staining, parapodia were rinsed in three quick changes and subsequently in two 10-min changes of PBS with 0.1 % Triton and one 10 min rinse in PBS without Triton. Stained and rinsed samples were dehydrated in isopropanol (2 min 70 %, 2 min 85 %, 2 min 95 %, 2 min 100 %, 2 min 100 %) and cleared in three 15-min changes of Murray Clear. The samples were placed in hollow-ground slides, mounted in Murray Clear, and sealed with nail polish.

The upper layers of musculature were partially removed from the confocal z stack, digitally, using Photoshop CS6 to allow viewing the chaetae within the torus. The entire CLSM image stack is available for download (link provided under data repository).

### Electron microscopy (TEM, SEM)

Specimens used for transmission electron microscopy were fixed using the same fixation method described above for semi-thin sectioning (1.25 % glutaraldehyde buffered in 0.05 M phosphate buffer with 0.3 M NaCl for 1.5–2 h, postfixation with 1 % OsO_4_ for 45 min). These specimens were also embedded in araldite and sectioned into a complete series of silver-interference coloured (70–75 nm) sections using a diamond knife (Diatome Histo Jumbo) on a Leica Ultracut S ultramicrotome. The serial section ribbons were placed on Formvar-covered, single-slot copper grids and stained with uranyl acetate and lead citrate in an automated TEM stainer (QG-3100, Boeckeler Instruments). The sections were examined using a Zeiss Libra 120 kV transmission electron microscope.

The chaetal formation was reconstructed using the information gathered from serial ultrathin sections and series of semi-thin sections of *S. alveolata*. The coverage of different stages of chaetogenesis was, with 14 consecutive developmental stages, dense enough to allow insights into the dynamics of the entire process that will be described in the following. The entire aligned stacks of ultra-thin and semi-thin sections are available for download (links provided under data repository).

For scanning electron microscopy (SEM) *Sabellaria alveolata was* fixed in Bouin’s fluid, dehydrated in an alcohol series and dried with CO_2_ in a critical point dryer (BALZERS). After dehydration the samples were sputtered with gold (BALZERS Sputter coater) and examined with a XL 30 SFEG (Philips Electron Optics) scanning electron microscope. During dehydration the animals were sonicated to remove debris and sand particles from the chaetae.

## Results

### Parapodial structure and chaetal arrangement

The body of *Sabellaria alveolata* is divided into four regions that are characteristic for Sabellariidae; the thorax, parathorax, abdomen, and the cauda (Fig. 1b). Chaetal elements in the thorax and parathorax comprise of opercular paleae, oar-shaped notochaetae and capillary chaetae. The abdomen of *S. alveolata* forms the largest part of the animal’s body and bears segmental biramous parapodia with notopodial uncini and neuropodial capillaries. The cauda has the appearance of an unsegmented tube and is achaetous. Aciculae are absent in all segments (Fig. 1b).

**Fig. 2:**
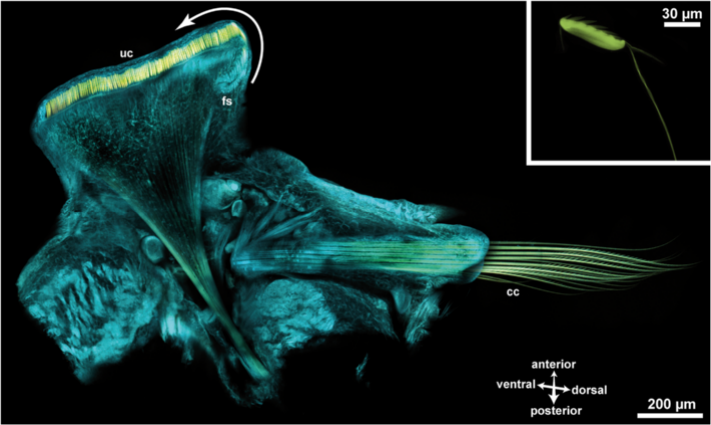
Confocal *z*-projection of a phalloidin stained preparation of a single abdominal parapodium of *Sabellaria alveolata. cyan* phalloidin*, yellow* chaetal autofluorescence *uc* uncini, *fs* formative site, *arrow* marks the direction of chaetal development *cc* capillary chaetae, *inset* detail image of an isolated uncinus

The abdominal notopodia are paddle-like appendages on either side of the animal’s body (Figs. 1b and 2). Those of the first few abdominal segments are broad and large, towards the posterior end they become narrower and elongate. Paired dorsal branchiae appear on the parathoracic segments and in the first 15–20 abdominal segments. They become gradually smaller along the antero-posterior axis and disappear completely in the posterior segments of the abdomen. The uncini are located at the apical margin, where they are aligned to form a single transverse row. Each chaeta arises from a chaetal follicle and all follicles are aligned within a single chaetal sac without being separated by an extracellular matrix.

**Fig. 3:**
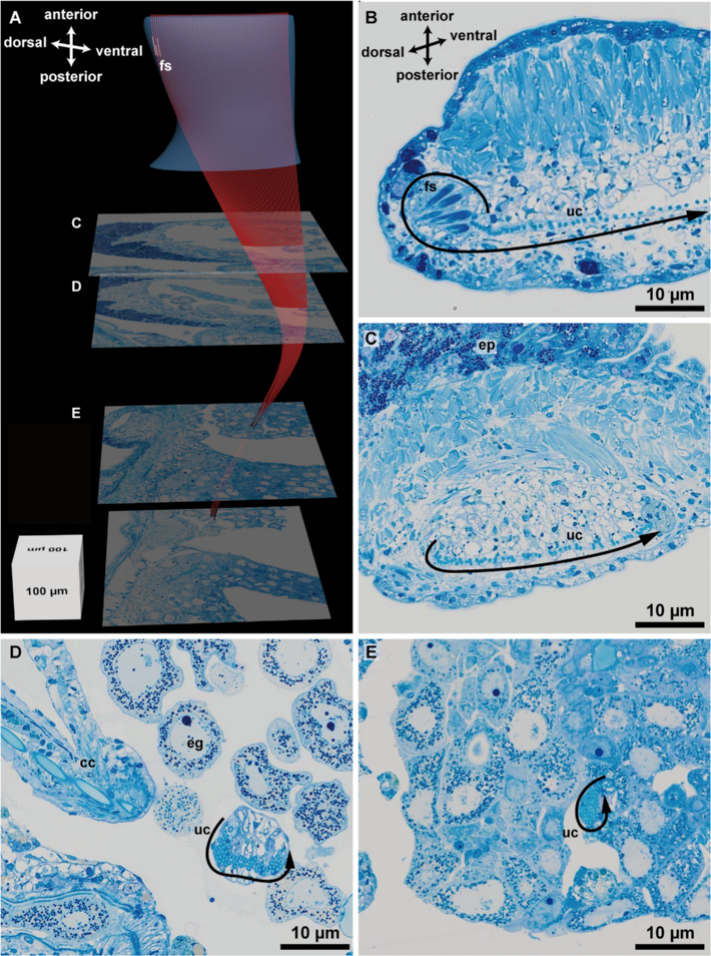
**a** 3D model of the chaetal arrangement inside an abdominal torus. **b**–**e** Aligned semi-thin sections used to construct the 3D model. Corresponding section planes are marked in A. The *arrow* indicates the direction of chaetal formation. *fs* formative site, *uc* uncini, *cc* capillary chaetae

Small, needle-shaped rods originate from the rostral and adrostal portion of each uncinus and extend into the notopodium. Apically these rods are aligned in a row, but as they reach deeper into the notopodium they form a bundle (Figs. 2, 3a). Each rod is surrounded by a follicle cell. The follicle cells of all rods comprise the inner end of the chaetal sac and rest on a common extra-cellular matrix (ECM). Follicle cells and ECM connect the bundle of rods to the parapodial musculature (see cLSM stack) in such a way that only the entire bundle can be moved, but not an individual chaeta. The formative site of the uncini is located at the ventral edge of the chaetal row and contains numerous developing chaetae (Figs. 2; 3a, b; 5a), so that chaetogenesis could be inferred in detail from an ultrastructural analysis of a series of different stages.

**Fig. 4:**
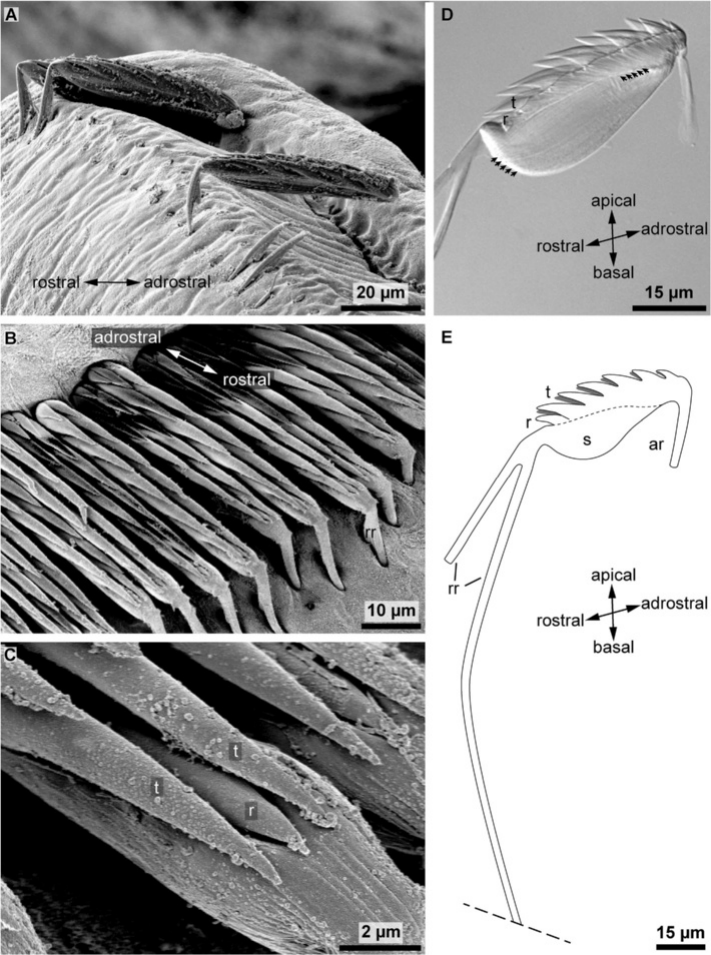
**a.** SEM image of detached abdominal uncini. **b** SEM image of the row of uncini. **c** SEM image showing the rostral portion of an uncinus in detail. **d** Micrograph showing the apical portion of an abdominal hooked chaeta. *arrows* mark the direction of the internal canals. **e** Schematic drawing of an abdominal uncinus, in scale. *dotted line* illustrates the refracting seam at merger of the chaetal socket and base, *rr* rostral rod, *ar* adrostral rod, *r* rostrum, *t* tooth, *s* socket

**Fig. 5:**
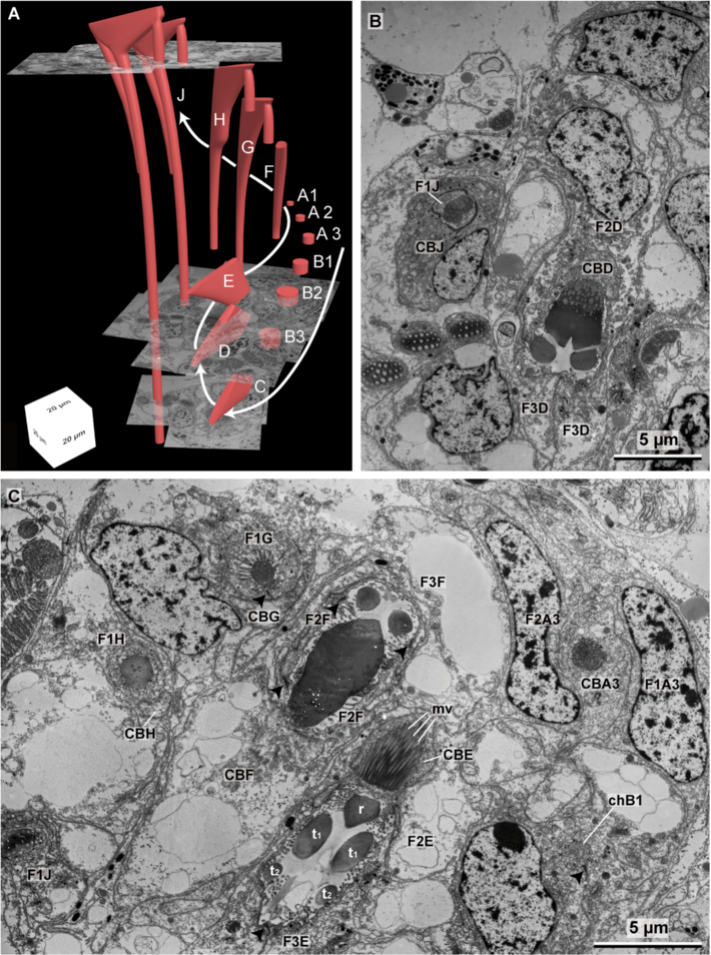
**a** 3D model of the chaetal formative site, reconstructed using the aligned serial ultra-thin sections. Consequent developmental stages of uncini are labeled from A1–J. This numbering is employed all through the images when referring to these specific developmental stages. **b** TEM image of the formative site showing the formation of the long rostral rod in J and the formation of the socket in D. **c** TEM image of the formative site showing the formation of the adrostral rod in H and G, the formation of the socket in E and F, and the formation of the rostrum in A3. Note the chaetal canal of B1 (*chB1*) in the lower right. *F1–F3* follicle cells, *CB* chaetoblast, *r* rostrum, *t* tooth, *arrow heads* mark the adluminal adherens junctions

The neuropodia of the abdomen only possess capillary chaetae that are either simple or pinnate (Figs. 2, 3d). Neuropodial capillary chaetae are long and also reach deeply into the parapodium. Their overall position is right-angled to the bundle of rods of the notopodial hooks (Fig. 2). The basis of the neuropodial chaetal sac is connected with a network of radial chaetal muscles to the outer body wall, giving the chaetae the characteristic arrangement similar to an arrow pulled back in a bow (Fig. 2). Upon contraction, these muscles shorten and push the chaetae out of the body surface.

### Structure of the uncini

Uncini in *Sabellaria alveolata* have a complex structure. The apical portion of a chaeta consists of a single median tooth followed by 5 to 6 pairs of teeth (Fig. 4d, e). The single small median tooth, called rostrum here, marks the rostral face of the uncinus; the size of the paired teeth decreases along a rostral-adrostral gradient, such that the adrostral pair of teeth is smaller than the rostral ones. All teeth and the rostrum originate from a blade-like shaft toward which they bend by 40°. Light microscopy shows that the shaft is composed of two different parts, separated by a fine rostro-adrostral refracting seam (Fig. 4d, e). The portion above this refracting seam directly underlies the teeth. It is small and dense, and we refer to this structure as the “base” hereunder. The portion of the shaft below the refracting line is keel-shaped and bright, and will be referred to as the “socket”. This socket has a length of ± 45 μm from rostral to adrostral. Under Nomarsky contrast, small, densely-packed vertical lines that originate in the teeth proceed into the base to end at the refracting seam. In the socket several lines can be seen running longitudinally and almost parallel to the refracting line. The main axes of both, vertical and longitudinal lines, form an angle of ± 40° (Fig. 4d). The teeth, the small underlying base and a tiny portion of the rostral rod are the only externally visible structures in SEM preparations (Fig. 4a, b). As mentioned above, each uncinus possesses two vertical rods, a shorter adrostral one and a bipartite rostral one. Soon after its origin the rostral rod splits into a short anterior and a long posterior rod. While the shorter (anterior) rostral rod is almost as long as the adrostral rod, the longer (posterior) rostral rod extends up to 1.5 mm deep into the notopodium. The posterior rostral rod is nearly 80 times longer than the entire apical portion (shaft plus teeth; ±20 μm) (Figs. 2, 4e). All anterior rods of the notopodial uncini form the above described intranotopodial fiber bundle that serves as the attachment site of notopodial muscles. Both rostral rods have a similar diameter (±1.5 μm) to that of the adrostral rod.

### Chaetogenesis

**Fig. 6:**
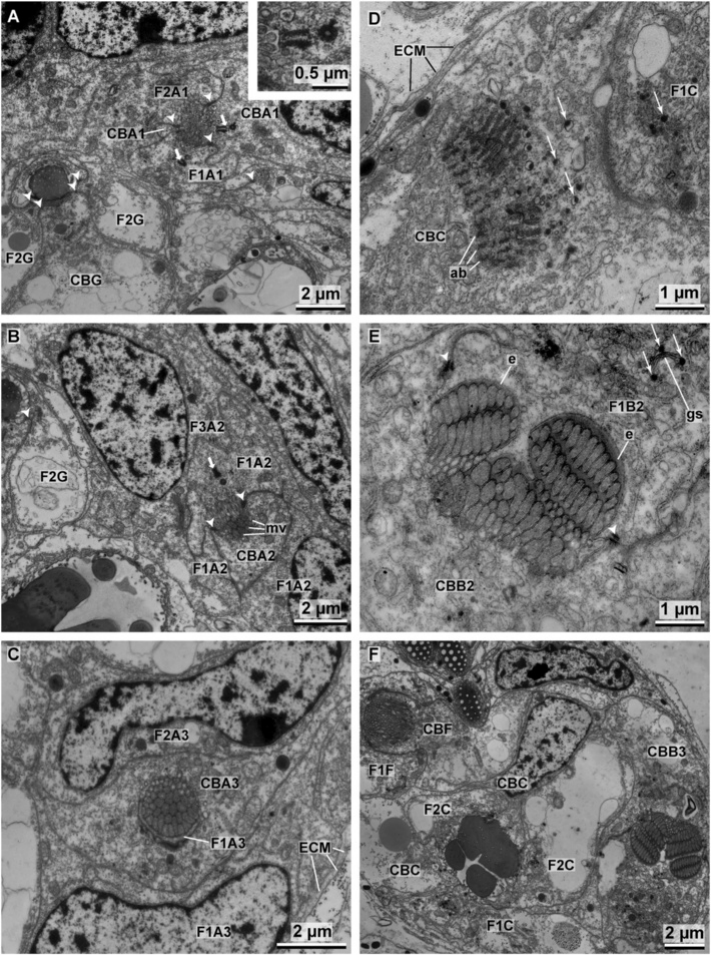
**a**–**c** TEM images of A1–3 showing the initial stage of chaetogenesis and the formation of a rostrum. *Inset* high magnification of a diplosome **d** Production of chaetal material and the subsequent transportation to the chaetal *anlage* via vesicles. **e** Formation of the adrostral teeth in B2. **f** TEM image of the formative site showing the formation of teeth in B3 with multiple rows of microvilli, older teeth in C with almost completely filled canals and the adrostral portion of the chaeta in F. *F1–F3* follicle cells, *CB* chaetoblast, *arrow heads* mark the adluminal adherens junctions, *short arrows* mark centrioles, l*ong arrows* mark vesicles containing electron-dense chaetal material, *ECM* extra-cellular matrix, *ab* actin bundles, *mv* microvilli, *e* enamel, *gs* golgi stack

**Fig. 7:**
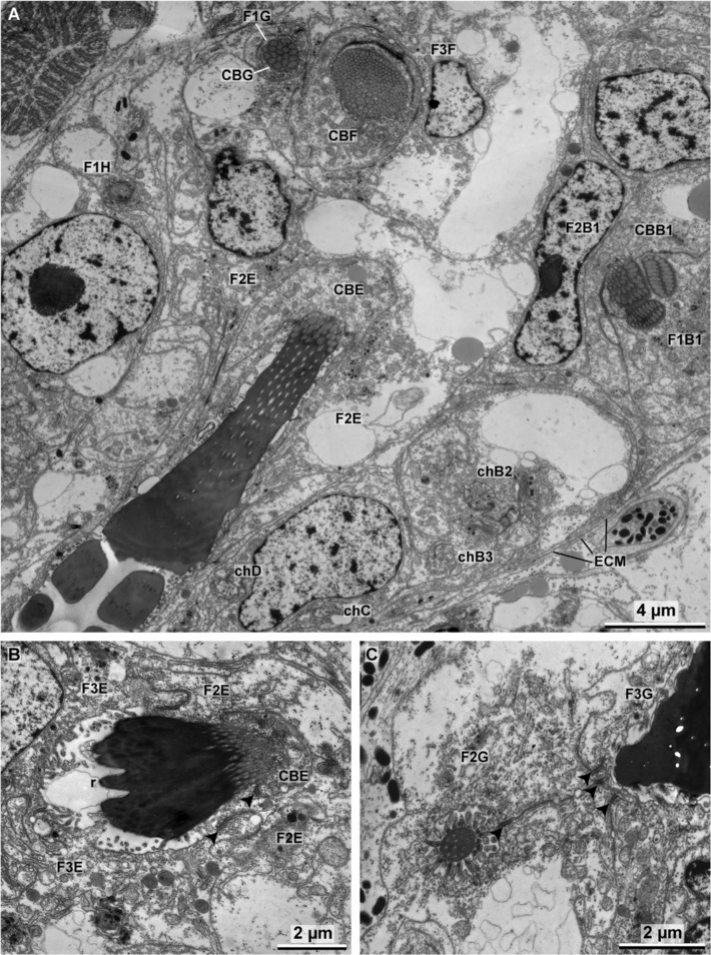
**a** TEM image of the formative site showing the formation of the short rostral rod in G, the formation of the subrostral portion of the socket in F, the formation of the socket in E and the formation of teeth in B1. Note the canals (*chB2–D*) that connect inferior developmental stages to the outer surface. **b** Formation of the rostral part of the socket in E. **c** Adrostral rod of G surmounted by F2. *F1–F3* follicle cells, *CB* chaetoblast, *arrow heads* mark the adluminal adherens junctions, *ECM* extra-cellular matrix, *r* rostrum

**Fig. 8:**
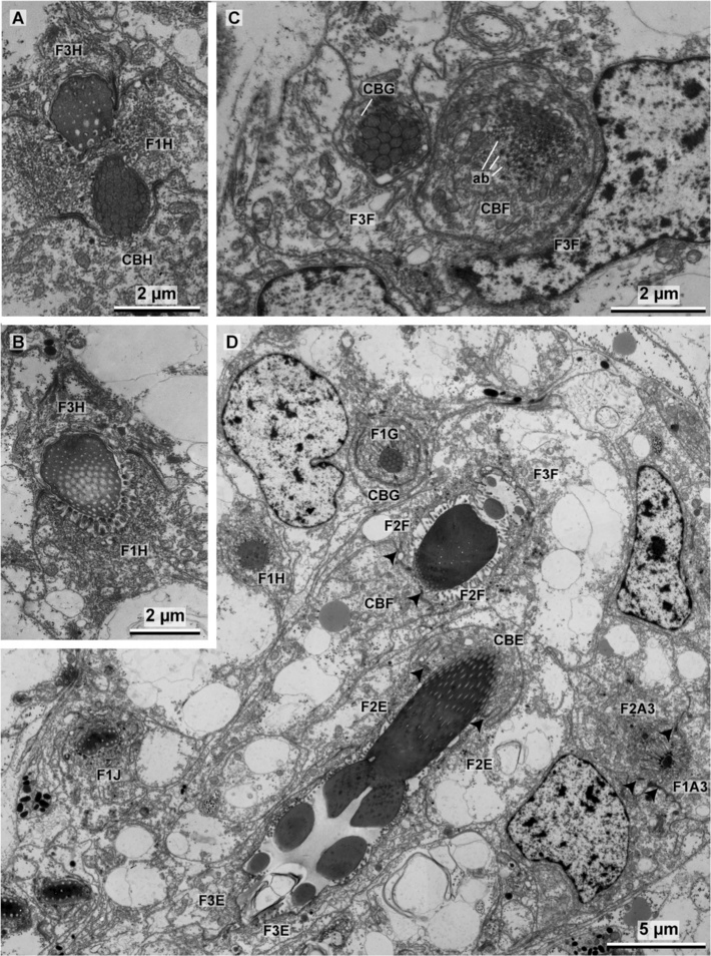
**a**–**b** TEM images showing the merger of the long and short rostral rod in H. Note the newly developing long rod and the fully differentiated short rod in A. **c** Formation of the short rostral rod in G and the formation of the subrostral process in F, note the bundles of actin filaments that are located under the microvilli. **d** TEM image of the formative site showing the fully differentiated long rostral rod in J, developing long rod of H, formation of the short rostral rod in G, formation of the socket in F–E, and the tip of the rostrums in A3. *F1–F3* follicle cells, *CB* chaetoblast, *arrow heads* mark the adluminal adherens junctions, *ab* actin bundles

Chaetogenesis occurs continuously within the formative site and the TEM study of fixed material allows inferring the entire process of chaetal formation from different developmental stages within a single formative site of the chaetal sac (Figs. 5, 6, 7 and 8). Uncini are formed within an ectodermal invagination (chaetal follicle) consisting of the chaetoblast and at least five follicle cells. All cells are epithelial, interconnected by adluminal adherens junctions (belt desmosomes) and septate junctions, and rest on a common matrix that surrounds the chaetal sac. All cells surround a small compartment, the chaetal compartment, and bear several short microvilli that reach into the compartment. This compartment narrows to become a small canal that extends towards the epidermis where it opens to the exterior by a small pore. During chaetogenesis the chaeta is secreted into the chaetal compartment. The basalmost four cells are actively involved in chaetogenesis, i.e. the chaetoblast at the base of the chaetal follicle and three adjacent follicle cells. The fourth and fifth follicle cells form a ring that surrounds the chaetal compartment and the proximal section of the canal. Each of these cells possesses a subapically located diplosome (Fig. 6a, b). In young follicles one of the two diplosomes may contact the apical cell membrane, but this never induces a cilium (Fig. 6a). The microvilli of the chaetoblast and the first two follicle cells are set more densely and are longer than those of the remaining follicle cells; the microvilli of the chaetoblast are slightly larger in diameter than those of the follicle cells (Fig. 6b). The latter form the template of each substructure of the chaeta. Continuous polymerization of chitin between the bases of the microvilli enlarges the developing chaeta. Given that the microvilli have a constant length, sooner or later the developing chaeta will exceed the microvilli in length and electron-lucent canals will remain inside the chaeta where microvilli had once been. These canals may be filled up secondarily by electron-dense material.

**Fig. 9:**
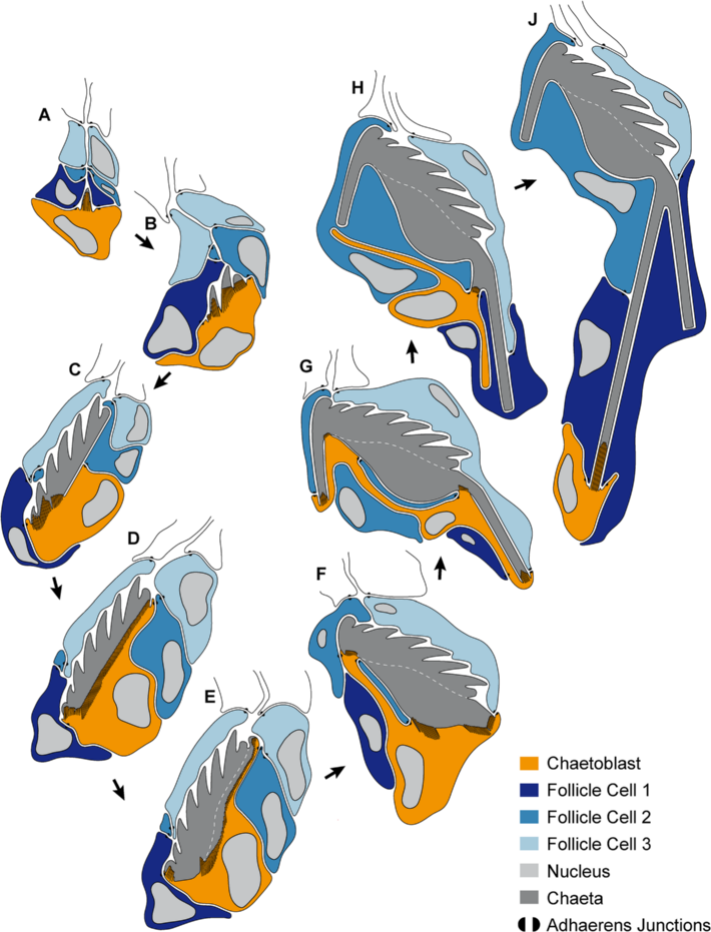
Schematic illustration of chaetogenesis and the interaction between the chaetoblast and the follicle cells as a series of sagittal sections of subsequent representative stages of the chaetal formation. Topological position of corresponding development stages are marked in the 3D model in Fig. 5. **a** Earliest stage of chaetogenesis; formation of the rostrum. **b**–**c** formation of the teeth. **d**–**e** formation of the chaetal socket. **f**–**g** Formation of the adrostral rod and the short rostral rod. **h**–**j** Final step of chaetogenesis; formation of the long rostral rod

In this study, 14 developmental stages of uncini were found in a single formative site that was cut into a series of ultrathin sections, analysed for ultrastructural details and reconstructed. Nine stages are shown in Fig. 9 and the topological position of these stages within the formative site can be seen in Fig. 5a. Chaetogenesis of uncini in *Sabellaria alveolata* can be divided into three steps: (1) formation of the rostrum, teeth and base (Fig. 9a–c), (2) formation of the socket (Fig. 9d–f), (3) formation of the rostral and adrostral rods (Fig. 9f–j).

**Fig. 10:**
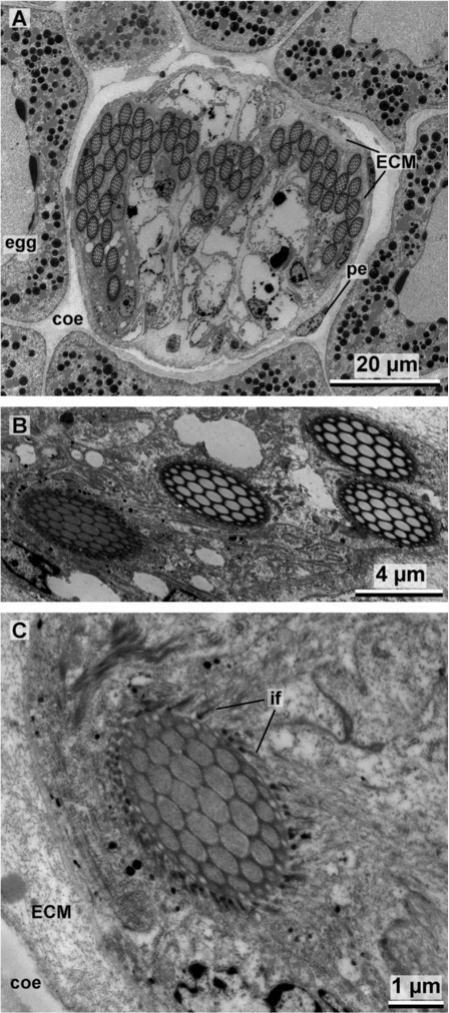
**a** TEM image of the chaetal bundle showing the arrangement of fully differentiated chaetae. **b** Canals of the youngest chaetae stilled filled with microvilli in contrast to the hollow canals of older chaetae. **c** Detail image of the youngest chaetae, note the intermediary filaments (*if*) attached to the chaeta via hemidesmosomes. *coe* coelom, *pe* peritoneum, *ECM* extra-cellular matrix

#### Formation of rostrum, teeth and base

Chaetogenesis begins when a small cluster of microvilli emerge on the surface of the chaetoblast (Figs. 6a–c, 9a). These microvilli form the template of the anteriormost tooth, the rostrum, and extend into the chaetal compartment. Chitin polymerizes between the bases of the microvilli and forms the tip of the rostrum. Additional microvilli that appear peripheral to the initial cluster broaden the rostrum. Subsequently, two additional clusters of microvilli are formed adrostrally on either side of the developing rostrum (Fig. 6d, e). These are the template of the first pair of teeth. In the same manner five additional pairs of teeth are subsequently added along a rostro-adrostral gradient, so that finally the sixth pair of teeth is situated adrostrally (Figs. 5c, 9b-c). All teeth and the unpaired rostrum have nearly the same size. Their templates were all once formed by 2–3 rows, each consisting of 9–12 microvilli. Since the number of microvilli increased towards the base of the teeth, all microvilli finally form a broad and uniform field, which is the template for the base underlying the teeth. Electron-dense material released from vesicles of the first two follicle cells forms an enamel that covers and smoothens the irregular surface of the teeth (Fig. 6e). This material is produced inside Golgi stacks and transported to the chaetal surface in vesicles (Fig. 6d, e). While more rows of teeth are added and the developing chaeta enlarges, the canals left by the templating microvilli, become more or less completely filled with electron dense material (Fig. 6f). At the end of this first step of chaetogenesis the rostrum, four pairs of teeth, and the anterior part of the base are formed. The microvilli are completely retracted from the rostral three quarters of the developing chaeta; the canals the microvilli left, are refilled with electron-dense deposits. The chaetoblast merely underlies the adrostral half of the developing chaeta, whereas the rostral half is underlain by the planar apical cell membrane of the second follicle cell. Chaetogenesis is interrupted in this region. The entire *anlage* is oriented vertically within the chaetal compartment.

#### Formation of the socket

Once all teeth and the base are formed, the chaetoblast grows towards the rostrum again to underlie the entire base and slightly exceed it rostrally. The chaetoblast then forms microvilli that form a homogeneous field These microvilli have a vertical orientation and thus are more or less longitudinal relative to the *anlage*. They form the template of the socket and chitin polymerizing between the microvilli is added to the base of the *anlage*. The longitudinal refracting seam visible under Nomarsky contrast between base and socket results from the break in chitin polymerization after teeth and base were formed (Figs. 5c, 7a,b, 8d, 9d-f). Re-orientation of the microvilli is also clearly visible under Nomarsky contrast in fully differentiated chaeta as longitudinally arranged lines inside the socket. These lines are actually canals left by microvilli inside the socket during formation (Fig. 4d, e). A group of microvilli remains at the apico-adrostral part of the chaeta, while those in the subapical part disappear. Bare cell membrane of the chaetoblast underlies this portion and no chitin is formed (Fig. 6f, 9e, f). At this time the entire *anlage* begins to alter its position within the chaetal sac again. Since the microvilli are always vertically oriented they also alter their position relative to the developing chaeta. The subapical group of microvilli forms an adrostral cap while the socket increases in size. Finally the microvilli that formed the template of the adrostral portion of the socket retract and disappear, except for those microvilli that formed the adrostral cap. The same occurs rostrally, here leaving a large apico-rostral group of microvilli (Fig. 9f). Adrostrally, the second follicle cell expands into the gap between the developing chaeta and the chaetoblast, so that this part of the developing chaeta is now underlain by the apical cell membrane of the second follicle cell. The subrostral portion of the socket is underlain by the apical cell membrane of the chaetoblast. After the socket has been completed two groups of microvilli remain, a rostral and an adrostral one. The entire *anlage* now has a horizontal position within the chaetal compartment (Fig. 9f).

#### Formation of the rostral and adrostral rods

After the socket is completed the microvilli of the chaetoblast are almost completely reduced, except for a rostral and an adrostral group (Fig. 9f). The adrostral group of microvilli elongates and forms the template for the adrostral rod. Chitin polymerization happens rapidly and the adrostral rod elongates, parallel to the apico-basal axis of the uncinus (Fig. 9g). The rostral group of microvilli actually consists of two adjacent, but perpendicular patches of microvilli (Figs. 7a, 8c). They form the template for the rostral rod, which initially is rather massive and oblique to the rostro-adrostral axis of the chaeta. Later the microvilli split into an anterior and a posterior one. The microvilli of the anterior group elongate and become the template for the anterior rostral rod, while the posterior group consists of short microvilli and remains in its original position. Chitin polymerizes rapidly between the microvilli to form the anterior rostral rod. Anterior rostral rod and the adrostral rod are formed simultaneously; one keeps pace with the other during formation (Fig. 9g). During these initial steps of forming the rods the chaetoblast expands as the anterior rostral rod grows slightly oblique to the apico-basal axis of the developing chaeta. The perikaryon of the chaetoblast is located rostrally and a small adrostral cytoplasmic bridge underneath the socket connects the perikayon to the adrostral group of microvilli (Figs. 5, 9g). A small rostral cytoplasmic bridge connects the rostral group of microvilli that is the template of the anterior rostral rod. After both are completed, the microvilli are reduced and the cytoplasmic bridges are withdrawn (Fig. 9h). The adrostral cytoplasmic bridge is replaced by the second follicle cell, which already grew between the median portion of the socket and the chaetoblast earlier during chaetogenesis (Fig. 9f). The rostral cytoplasmic bridge is replaced by the first follicle cell. During withdrawal the last group of microvilli which remained posterior while the anterior rostral rod was formed, becomes active. Its microvilli elongate and form the template of the posterior rostral rod (Figs. 5b, 9j). While chitin polymerisation elongates the rod, the chaetoblast forms a cup that surrounds the developing posterior rostral rod. The posterior rostral rod increases very rapidly in length and grows parallel to the baso-apical axis of the chaeta (Fig. 10). No further modification of the microvilli pattern occurs in this last phase of chaetogenesis. During elongation the newly formed chaeta is pushed towards the surface, and finally becomes visible externally and aligns itself at the ventral edge of the chaetal row. The canals left by the microvilli during growth of the posterior rostral rod are not filled by any material and remain electron-translucent. The same is true for the anterior rostral rod and the adrostral rod. When the formation is complete, intermediate filaments appear inside the follicle cells and the chaetoblast. Hemidesmosomes connect them to the chaeta to mechanically link the chaeta to the perifollicular ecm (Fig. 10c). The chaetoblast remains cup-like at the chaetal base and the microvilli that formed the long rostral rod remain inside the basalmost part of the chaeta (Fig. 10b, c).

## Discussion

Uncini have repeatedly been described and illustrated for different sabellariid species [23–26], a practice that supports the adoption of *Sabellaria alveolata* as a representative for the entire group. Due to structural similarities of these chaetae across sabellariids we also assume that formation of them is largely identical in sabellariid species. However, the tremendous length of the posterior rostral rod has remained largely unnoticed. We suppose that this is caused by its extremely delicate structure, making it difficult to identify the actual extension of this rod without sectioning. Despite missing evidence from other species, we assume that a rostral rod extending deeply into the notopodium is characteristic for all sabellariid species, an assumption that has to be confirmed in subsequent studies. In the following we discuss our results in terms of function, homology, and phylogenetic significance, and highlight the cellular dynamics underlying chaetogenesis in *Sabellaria alveolata*.

### Function

It has repeatedly been shown that hooked chaetae and uncini correlate with a tubiculous lifestyle and are used to withstand drag forces by interacting with the inner texture of the tube [27, 28]. Roy [29] reports that the sabellariid *Phragmatopoma californica* maintains its position in the center of the tube by extending the notopodia so that they contact the wall. Thereby, the notopodia must be of a certain length to maintain water currents inside the tube for oxygen supply and feces removal. We assume that the uncini serve as anchors to adhere to the visco-elastic wall of the tube [30]. In *Sabellaria alveolata* a second function is related to these chaetae, the structural correlate of which is the rods. The posterior rostral rod is 80 times longer than the shaft and extends deeply into the neuropodium. A rod consists of a few hollow chitin tubes wrapped in an enamel and serve as a rigid, but extremely flexible stick. Since the uncini of *S. alveolata* are aligned in a transverse row, these rods form a planar array in the tip of the notopodium that thus serves as a broad paddle and allows a maximum contact surface between the row of uncini and tube wall. Deeper inside the notopodium the rods converge to form a bundle that serves as the attachment site for parapodial muscles. The notopodium contains part of the body coelom, which functions as a hydroskeleton that guarantees the stiffness of the notopodium, but does not allow mobility. Since the bundle of rods is highly flexible and serves as an attachment site for transversal muscles, the notopodium can be moved backward and forward. Due to the mechanical properties of the chitinous rods inside, it will always return to its original structure after relocation. The rods thus serve in stability of the notopodium and allow moving it without influencing the shape of the notopodium. Roy [29] actually mentions that *Phragmatopoma californica* uses the notopodia to perform rear-to-front motions. These anteriorly directed strokes are used for backward moving when the animal rapidly withdraws into the tube. The structural prerequisite of such a notopodial performance is the internal bundle of rods. In this respect the bundle of rods in sabellariid notopodia has a similar function as the acicula of errant (aciculate) annelids. The aciculae also function as “skeletal” rods of parapodia to which the parapodial musculature is attached. In certain terebellids (i.e. *Terebella lapidaria*) similar long shafts/basal processes reach deep inside the parapodia (unpublished data). This indicates a convergent evolution of rod-like elements inside the parapodia, be it bundles of thin rods like in sabellarids, other chaetal protrusions like in terebellids or large and robust aciculae.

### Homology

According to Holthe [31] hooked chaetae (= dentate hooks in Rouse & Plejel [32]) consist of a main tooth or rostrum, a capitium surmounting the rostrum, and smaller teeth and a manubrium or shaft. The rostrum and teeth of the capitium are curved and bend towards the shaft. Sometimes a subrostral process or distal expansion of the manubrium is found underneath the rostrum. Such chaetae are known from Sabellida, Terebellida, Oweniidae, adult Arenicolidae [11, 12, 15, 18]. If the shaft is shorter than the dentate distal section or virtually absent, the hooked chaetae will be called uncini (Sabellida, Terebellida, Chaetopteridae, Sabellariidae) [32]. In certain groups the dentate apex (rostrum plus capitium) is partly enveloped by hair-like protrusions of the subrostrum (juvenile Arenicolidae, Maldanidae, Psammodilidae) [10, 15, 16] or a hood (Capitellidae, Spionidae, certain Eunicida) [9, 33, 34]. It is thus unsurprising that testing for homology by studying chaetogenesis has revealed that hooked chaetae and uncini of certain taxa share identical steps during chaetogenesis (Sabellidae and Serpulidae: [11] Arenicolidae: [14, 15], Maldanidae: [10], Psammodrilida:[16], Pectinariidae: [12] , Terebellidae: [17], Oweniidae: [18]). In these taxa, the rostrum is always the very first structure that develops during genesis and is invariably preformed by a group of microvilli. Subsequently, each teeth of the capitium are formed by a large microvillus. Microvilli that served as template for the rostrum and the capitium later merge and form the shaft, which always is perpendicular to the rostrum. These characteristics are found in capitellid’s hooded hooks, i.e., chaetae, in which a hood surrounds the distal section of the hooked chaeta [33]. The identity of the structure and formation patterns of hooked chaetae, uncini and hooded hooks across the above mentioned annelid taxa led to the hypothesis of their homology, which could be substantiated by several corresponding structural and developmental details [4, 17]. Other hooked chaetae with a hood differ from this pattern. In spionid and lumbrinerid species several microvilli and not a single microvillus form the template for the smaller spines that surmount the rostrum (for *Scolelepis squamata* [34]; for *Prionospio fallax* [35], for *Lumbrineris tetraura* [9]). In addition formation of the hood differs between spionid, capitellid and lumbrinerid species and does not support homology of the hood [9].

The structure and chaetogenesis of uncini in *Sabellaria alveolata* differ significantly from any hooked chaeta described so far, which poses problems in the application of terminology. Although the unpaired rostral tooth should be termed the rostrum, as it is the first structure formed during chaetogenesis and preformed by several microvilli, further groups of microvilli serve as the template for the following teeth, which thus should not be termed capitial teeth. The shaft consists of two sections, the base and socket; both are separated by a refracting line and are pre-formed by microvilli of different orientation. A shaft that is composed of two parts because of controlled spatial and temporal intermissions in the formation processes is thus far unknown. The subcuticular portion of sabellarid uncini consists of an adrostral and a bipartite rostral rod. Similar rod-like processes are known from terebellids (*Nicolea zostericola*; [17]) and chaetopterids (Tilic & Bartolomaeus, unpubl. data for *Chaetopterus variopedatus* and *Telepsaphus costarum*). However, the formation of these processes differs from the rods in *Sabellaria alveolata.* Two small groups of microvilli, one rostral and one adrostral remain in the mentioned terebellid and chaetopterid species after the microvilli were withdrawn from the shaft after its completion. Polymerization of chitin between the microvilli of both groups then gives rise to both processes, which thus are rather parts of the shaft than additional structures. These differences do not support a homology between the rod in *Sabellaria alveolata* and the rod-like processes of the manubrium in terebellid and chaetopterid species. Structure and chaetogenesis of uncini in *S. alveolata* thus differ in several aspects from that of other annelids with uncini and hooked chaeta. These differences either result from transformation or convergent evolution. A decision between both alternatives, however, depends on the phylogenetic position of Sabellariidae.

### Phylogenetic implications

Sabellariidae were first described as a subgroup of Sabellida by Lamarck [36], and later moved to Terebellida by Savigny [37]. Levinsen [38] placed them as a separate suborder, using the name Hermelliformia, which was first coined by Malmgren [39]. Phylogenetic analyses based on morphological data [40–42] suggest a sister group relationship with Sabellidae. One decisive morphological character in favour of the close relationship to Sabellidae is the so-called “chaetal inversion” (for review [43]; [44–48].) Sabellaridae and Sabellidae show a unique chaetal arrangement with abdominal uncini in a notopodial position. This was considered to be an undisputed synapomorphy until Kieselbach and Hausen [43] provided evidence that the specific chaetal arrangement of Sabellidae and Sabellariidae arose independently (see also [49]). Kieselbach and Hausen [43] also emphasize that the homology of the uncini of sabellids and those of sabellarids is yet to be established. More recent molecular phylogenies of annelids [47, 48, 50–54] group them together with Spionida. A sabellariid-spionid sister group relationship had already been suggested by Wilson [55] and later Dales [56] and Rouse & Pleijel [32, 57]. Wilson [55] substantiated this hypothesis with characters of the larval organisation, since both possess long larval chaetae inserting posterior to the prototroch. Kieselbach [49] described a specialized ciliated sensory organ in the prostomium, of larval *Sabellaria alveolata* that was thus far only known from Spionida [58]. Except for being paired in Spionida, this organ shows an identical organization and the same substructures like Spionida, so that this sense organ supports the hypothesis of a sister group relationship of Spionida and Sabellariidae.

The differences of sabellid and sabellariid uncini in terms of substructures and chaetogenesis, however, do not provide evidence for a sister group relationship between both groups. Moreover, the fact that several microvilli and not a single big microvillus form the template for each adrostral teeth is identical in the spionids studied thus far [34, 35] and in *S. alveolata*. The better supported alternative hypothesis of a spionid-sabellariid sister group relationship presently argues against homology of sabellid and sabellariid uncini and for transformations that need to be analysed in subsequent studies.

### Cell dynamics

In a recent essay Warren [59] compared the microvilli of the chaetoblast with the printing head of a 3D-printer, as they ensure assembly of a complex structure by selective addition of material in time and space. Chaetogenesis in *Sabellaria alveolata* illustrates the complexity of this process and provides empirical evidence that in addition to dynamic microvilli cell dynamics influences proper formation of the chaeta. Beside repeated formation of microvilli, the position of the chaetoblast within the formative site and the speed in which chitin polymerizes are important factors shaping the final structure of the sabellariid uncinus. Tilting the axis of the developing chaeta is a prerequisite to form the basal part of the shaft, the socket, as well as the proper orientation of the rods. The chaetoblast itself expands during chaetogenesis, relocates the perikaryon and finally remains as a cup-like structure at the base of the rostral rod. During this final step of chaetogenesis the follicle cell expands tremendously as it surrounds the entire posterior rostral rod. Since the rod, when completed is 80 times longer than the shaft, the follicle cell expands to 80-fold of its initial length.

Fixation of a continuous developmental process causes that this process is divided into different stages. The formative site of *S. alveolata* studied in this paper, shows 13 of these stages (Fig. 5a). Provided that chaetogenesis is a continuous process, one would expect that the time passed between the stages is always identical, even though it is not exactly known. According to this consideration, the initial phase of chaetogenesis lasts rather long, since we found six subsequent stages showing increasing numbers of apical teeth. The remaining steps are rather rapid events, because six steps later the entire chaeta is complete, except for the posterior branch of the rostral rod. One step further this structure attained an enormous length. Chitin is produced by the chitin synthases that are located in the cell membrane and has been shown to appear at the bases of microvilli [60–62]. Provided that chitin synthase is also located in microvillar membrane, one would expect that the longer the microvilli are the higher is the rate of chitin synthesis. Although this remains to be shown experimentally, there is a remarkable correlation between the length of the microvilli and the speed of growth of chaeta in support of this anticipation: the longest microvilli can be found where chaetal elongation occurs rapidly.

## Conclusions

Despite superficial similarities to the uncini of Sabellida, Terebellida and other smaller annelid groups, the uncini of *S. alveolata* differ in substructures and formation (Table 1). These differences concern (1) formation of adrostral teeth by groups of microvilli instead of one large microvillus, (2) bipartition of the shaft and its formation in temporally separated steps and (3) formation of rostral and adrostral manubrial extensions (4) followed by the formation of an adrostral and a bipartite rostral rod. These differences either result from transformations of an ancestral structure or from convergent evolution. Given that recent molecular and morphological data provide strong support for a sister group relationship between Spionida and Sabellariidae, the uncini in sabellariids on one hand and those of terebellids, sabellids and a few other smaller annelid taxa on the other hand appear to have evolved convergently. Since Spionidae possess hooded hooks consisting of apically dentate chaeta with a hood and since all apical teeth are pre-formed by groups of microvilli, it is likely that the sabellariid uncini evolved by transforming such dentate chaetae into uncini. Our study also shows that this transformation went along with changing functional demands. In contrast to spionid species, sabellariids live in a reinforced visco-elastic tube to which they are able to firmly adhere, using the uncini as anchors. The specific structure of the notopodium optimizes the contact surface towards the tube wall. In addition the notopodia are used for rapid withdrawal and must be movable. Since they are rather long structures they need some internal reinforcement that acts as attachment site for the transversal muscles. These attachment sites are provided by the rods originating from the uncini, since they form a central, flexible structure comparable to the acicula in aciculate annelids.

**Table 1:** Structure and chaetogenesis of hooked chaetae and uncini in Annelids

Taxon	Species	Substructure								Formation		Reference
		Rostrum/main tooth	Adrostral teeth	Capitium	Shaft bipartite	Shaft length	Hood	Beard	Basal extensions	Rostrum	Adrostral teeth	
										Microvilli	Microvilli	
Sabellariidae	*Sabellaria alveolata*, abd	+	+	-	+	s	-	-	r	several	several	this study
Spionida	*Scolelepis squamata*	+	+	-	-	l	+	-	-	several	several	Hausen & Bartolomaeus 1998 [34]
	*Malacoceros fuliginosus*	+	+	-	-	l	+	.	-	several	several	Hausen & Bartolomaeus 1998 [34]
	*Prionospio fallax*	+	+	-	-	l	+	-	-	several	several	Hausen 2001 [35]
	*Spirorbis spirorbis*, abd	-	+	+	-	s	-	-	-	-	single	Bartolomaeus 1995 [12]
Sabellida	*Fabricia stellaris*, tho	+	+	+	-	l	-	-	-	several	single	Bartolomaeus 2002 [11]
	*Fabricia stellaris*, abd	-	+	+	-	s	-	-	-	-	single	Bartolomaeus 2002 [11]
	*Branchiomma bombyx*, abd	+	+	+	-	s	-	-	-	several	single	unpubl. data
	*Pseudopotamilla reniformis, abd*	+	+	+	-	s	-	-	-	several	single	Kolbasova et al. 2014 [13]
Terebellida	*Pectinaria koreni*	-	+	+	-	s	-	-	-	-	single	Bartolomaeus 1995 [12]
	*Pectinaria auricoma*	-	+	+	-	s	-	-	-	-	single	Bartolomaeus 1995 [12]
	*Nicolea zostericola*	+	+	+	-	s	-	-	p	several	single	Bartolomaeus 1998 [17]
Chaetopteridae	*Telepsaphus costarum*	-	+	+	-	s	-	-	p	-	single	unpubl. data
	*Chaetopterus variopedatus*	-	+	+	-	s	-	-	p	-	single	unpubl. data
Arenicolidae	*Arenicola marina*, juvenile	+	+	+	-	l	-	+	-	several	single	Bartolomaeus & Meyer 1997 [15]
	*Arenicola marina*	+	+	+	-	l	-	+	-	several	single	Bartolomaeus & Meyer 1997 [15]
Maldanidae	*Clymenura clypeata*	+	+	+	-	l	-	+	-	several	single	Tilic et al. 2015 [10]
	*Johnstonia clymenoides*	+	+	+	-	l	-	+	-	several	single	Tilic et al. 2015 [10]
Psammodrilidae	*Psammodrilus balanoglossoides*	+	+	+	-	l	-	+	-	several	single	Meyer & Bartolomaeus 1997 [16]
Capitellidae	*Capitella capitata*	+	+	+	-	l	+	-	-	several	single	Schweigkofler et al. 1998 [33]
Oweniidae	*Owenia fusiformis*	-	+	+	-	l	-	-	-	-	single	Meyer & Bartolomaeus 1996 [18]
Lumbrineridae	*Lumbrineris tetraura*	+	+	-	-	l	+	-	-	several	several	Tilic et al. 2014 [9]

*abd.* abdomen, *tho.* thorax, *+* present, − absent, *s* short, *l* long, *r* rods, *p* processes

### Data repository

To allow full transparency of the data presented in this study, all of the aligned serial semi-thin and ultra-thin sections and the confocal z-stack of the phalloidin stained parapodium are freely accessible in the morphological database, MorphDBase: www.morphdbase.de [63].

Complete series of aligned ultra-thin sections:

Direct link: www.morphdbase.de/?E_Tilic_20151015-M-27.1

Complete series of aligned semi-thin sections:

Part 1– Direct link: www.morphdbase.de/?E_Tilic_20151015-M-29.1

Part 2 – Direct link: www.morphdbase.de/?E_Tilic_20151015-M-28.1

Confocal z-stack of the phalloidin stained parapodium:

Direct link: www.morphdbase.de/?E_Tilic_20151015-M-30.1
